# Exploratory Genome and Transcriptome-Wide Association Analyses of Addiction-Related Phenotypes in a Twin Cohort

**DOI:** 10.3390/biomedicines14081677

**Published:** 2026-07-26

**Authors:** Jiahua Zhou, An Phuc Ta, Catherine Yang, Ahmed El Shamy

**Affiliations:** 1 College of Medicine, California Northstate University, Elk Grove, CA 95757, USA; jiahua.zhou9513@cnsu.edu (J.Z.); anphuc.ta@gmail.com (A.P.T.); 2 College of Graduate Studies, California Northstate University, Elk Grove, CA 95757, USA; catherine.yang@cnsu.edu

**Keywords:** assortive mating, behavioral disinhibition, functional genomics, mesolimbic system, polygenic risk, pleiotropy, externalizing spectrum, transcriptome-wide association study

## Abstract

**Background/Objectives:** Substance use behaviors share a complex, overlapping polygenic architecture, yet translating genome-wide association study (GWAS) findings into actionable biological mechanisms remains challenging. This study aimed to characterize the genetic architecture of five substance use traits (alcohol consumption, alcohol dependence, nicotine use, illicit drug use, and behavioral disinhibition) and identify shared and distinct gene expression signatures within the neural circuits governing addiction. **Methods:** We reanalyzed 7188 individuals from the Minnesota Center for Twin and Family Research (MCTFR) cohort utilizing longitudinal composite phenotypes spanning five substance-use domains and general behavioral disinhibition. Post-QC, 6874 individuals were retained for downstream analysis. Following genomic imputation and linear mixed model GWAS (GEMMA), we utilized the SNipar framework to partition polygenic risk scores (PRS) into direct and indirect genetic effects, investigating intergenerational shifts in genetic penetrance and effects of assortative mating. Finally, we integrated our summary statistics with brain tissue reference panels to perform a transcriptome-wide association study (TWAS) modeling genetically regulated gene expression within neural circuits relevant to addiction. **Results:** Partitioning of polygenic risk revealed that while surface-level parental DNA correlations were modest (r = 0.08), underlying latent genetic correlations approached unity (R_δ_ ≈ 0.99), indicating that addiction risk clustering in families is driven by intense assortive mating and concentrated biological inheritance. Multi-phenotype TWAS identified several significant gene–phenotype associations—notably *ADAM32* and *SLC9A3*, which demonstrated pleiotropic effects across multiple substance use categories. Crucially, these significant TWAS signals were enriched in striatal structures (caudate, putamen, substantia nigra) and frontal cortical regions. **Conclusions:** Our findings support a model of shared genetic liability across diverse substance use behaviors, mediated by specific gene expression patterns in the mesolimbic dopamine system and frontal cortex. By integrating multi-phenotype GWAS and TWAS, this study highlights pleiotropic candidate genes and provides critical insights into the tissue-specific neurobiological pathways underlying addiction vulnerability.

## 1. Introduction

Substance use disorders and behavioral disinhibition are highly heritable conditions characterized by altered reward circuitry and executive function. These disorders frequently present concurrently and share a highly polygenic architecture. Recent massive genome-wide association studies have identified numerous loci shared across multiple substance use disorders, pointing to a generalized addiction risk factor [1,2]. However, despite these genomic advances, the field continues to struggle with the missing heritability problem, wherein the variance explained by common single nucleotide polymorphisms (SNP) falls far short of the total heritability estimated from classical family studies [3,4].

A critical limitation contributing to this gap is the reliance of massive biobanks on minimal phenotyping to maximize sample sizes. This breadth over depth approach often misses the nuanced dimensional traits, intergenerational dynamics, and longitudinal variance captured by family and twin designs [3]. Furthermore, standard population studies frequently suffer from population stratification and unmeasured environmental confounds, whereas familial biometric models inherently control these issues and capture total heritability [5,6].

To bridge this gap, we reassess deeply phenotyped transdimensional data from a previously published family-based, twin cohort—Minnesota Center for Twin and Family Study (MCTFR). We employ exact linear mixed models via the GEMMA toolset to efficiently account for familial relatedness and integrate highly powered polygenic risk scores (PRS) from external biobanks, using familial phenotypic depth to capture generalized genetic liability [1,7]. This familial framework provides control for population stratification and allows us to evaluate developmental moderation across parent and offspring generations, as well as characterize direct and indirect effects (i.e., the effect of an allele in the individual on the individual versus the effect of an allele in a parent on the offspring mediated by the environment, respectively) missing in traditional GWAS pipelines [8,9].

To translate these statistical genetic discoveries into biological mechanisms, we performed a transcriptome-wide association study (TWAS). By integrating our summary statistics with specific brain tissue expression panels, this approach models genetically regulated gene expression to identify candidate genes and distinct neural circuits that mediate addiction risk [10,11].

## 2. Materials and Methods

The discovery dataset was drawn from the Minnesota Center for Twin and Family Research (MCTFR), a longitudinal family study of adolescent twins, adoptive and biological siblings, and their parents. Participants completed comprehensive follow up assessments approximately every three years. The present analysis utilized 7188 individuals of European ancestry from this cohort (Accession: phs000620.v1.p1), who were genotyped using the Illumina Human660W Quad array (Illumina, Inc., San Diego, USA). Clinical phenotypes were derived utilizing a hierarchical factor analytic approach applied to data collected via the Substance Abuse Module of the Composite International Diagnostic Interview [12,13]. By integrating dimensional items spanning the frequency and quantity of use alongside strict DSM IIIR and DSM IV diagnostic symptoms, this process successfully constructed five continuous intermediate phenotypes: alcohol consumption, alcohol dependence, nicotine use, illicit drug use, and non-substance-related behavioral disinhibition [14,15].

To independently validate our results, we utilized data from the family-based, twin cohort from NIDA Australian Nicotine Addiction Genetics and Correlates study (Accession: phs001299.v2.p1). This confirmatory cohort consisted of 3508 individuals of European ancestry genotyped via a Smokescreen Affymetrix array (Thermo Fisher Scientific, Santa Clara , USA). This dataset provided biologically correlated phenotypes evaluated through categorical DSM diagnostic criteria. Utilizing this secondary cohort allowed us to robustly validate our primary continuous dimensional findings against established dichotomous clinical definitions.

Genotype data were quality controlled using PLINK v1.9. Individuals and variants with >1% missingness were excluded, variants with minor allele frequency <1% were removed, and deviations from Hardy–Weinberg equilibrium were filtered at *p* < 1 × 10^−7^. Sex discrepancies were evaluated using X-chromosome heterozygosity checks. Analyses were conducted on autosomal chromosomes (1–22). Post-QC, 6874 individuals in the MCTFR cohort were retained for downstream analysis.

Cleaned genotype data were submitted to the Michigan Imputation Server for pre-phasing and imputation using Eagle v2.4 with the HRC reference panel. Quality control statistics indicated complete overlap with the reference panel (100% reference overlap) and no allele inconsistencies, including zero allele mismatches, strand flips, or allele switches. No variants were removed due to invalid alleles, multiallelic status, duplication, non-SNP classification, monomorphism, or low call rate (<90%). A total of 409,746 variants passed quality control and were retained for imputation. Twelve variants were identified as typed-only sites. One genomic chunk containing fewer than 20 SNPs was excluded by the imputation pipeline, resulting in 139 chunks proceeding to imputation.

We utilized the phenotypes derived from a hierarchical factor analytic approach, focusing on a five-factor model of addiction liability. This framework builds upon extensive longitudinal assessments establishing behavioral disinhibition as a latent precursor to poly-substance use disorders done by prior MCTFR researchers [12,13].

To identify genetic variants associated with addiction phenotypes while accounting for complex familial relatedness, we utilized the GEMMA toolset [7]. This implements an exact linear mixed model (LMM) that is superior to standard regression for structured family data as it accounts for cryptic relatedness [16]. We utilized Likelihood Ratio Test (LRT) to generate *p*-values to ensure high sensitivity for detecting variants under structured conditions while mitigating false discovery rate inflation (FDR) [17,18].

A centered genetic relatedness matrix was constructed from all quality-controlled variants to capture shared environments and population stratification. We then performed univariate linear mixed model analyses independently for each of the five addiction scores using GEMMA. Each SNP was tested for association adjusting for the genetic relatedness matrix as a random effect, alongside fixed covariates including age, sex, generation, and top 10 principal components.

Finally, because substance use disorders are highly correlated, we conducted two distinct methods to evaluate for pleiotropy. Firstly, we conducted a multivariate linear mixed model analysis to jointly model variances across all traits simultaneously to detect variants underlying a generalized addiction liability [19]. Secondly, we identified variants that independently reached a suggestive threshold ($p<1\;\times \;10^{-5}$) in at least two out of the five univariate phenotype regressions.

We utilized two *p*-value thresholds for categorizing significant variants: a suggestive level ($p\;<\;5\;\times \;10^{-6}$) and a standard significance level ($p\;<\;5\;\times \;10^{-8}$) [20]. Variants meeting these thresholds were pruned for linkage disequilibrium using PLINK (v1.9) clumping with LD threshold of r^2^ ≥ 0.1, within a ±250 kb window around each index SNP, to retain the most significant variant per locus. This procedure yielded a set of 82 suggestive and 2 significant variants for downstream annotation using Ensembl Variant Effect Predictor (VEP; GRCh37 assembly). For each variant, the most severe predicted consequence was retained. Gene symbols and functional descriptions were extracted from Ensembl. rsIDs were assigned based on Ensembl variation databases; for variants lacking dbSNP identifiers, chromosomal coordinates (chr:position) were retained. Previously reported associations were queried from the NHGRI-EBI GWAS Catalog using the GWAS Catalog REST API. Variants were classified as having prior GWAS evidence if an association was reported for the corresponding rsID in the catalog. Reported traits and PubMed identifiers were recorded. All summary statistics were harmonized to ensure consistent allele orientation and genomic coordinates. Variants with missing or non-numeric *Z*-scores were excluded.

In the suggestive variant group, all 82 independent variants were tested in the validation sample using a quasi-likelihood score test (MFQLS) within the WISARD software (v1.3.2) package [21]. MFQLS conducts regression analyses tailored for dichotomous data while accounting for complex within-family genetic architecture and relatedness, using non-directional test statistics. To account for multiple testing, Bonferroni-corrected significance threshold was established at $p<6.1\times 10^{-4}$.

To address the missing heritability problem, we utilized our family-based data to examine how a person’s genetic vulnerability profile (represented via polygenic risk scores, or PRS) manifests through both direct and indirect effects. We aimed to bridge the gap between twin-study heritability estimates and the variance captured by traditional molecular-level associations with the GWAS and PRS pipeline approaches. To address biases from assortative mating and environmental confounding, we utilized the SNIPar toolset. By integrating Mendelian imputation techniques derived from Relatedness Disequilibrium Regression, SNIPar allowed us to recover missing parental data and isolate direct genetic effects more accurately [5,6,8,9]. For full methodology of this pipeline, please see the papers cited. The final hierarchical model demonstrated excellent fit to the observed clinical symptoms (CFI = 0.96, TLI = 0.95, RMSEA = 0.05), with all dimensional items loading significantly (lambda > 0.60) onto their respective latent factors.

Genetic liability was indexed using summary statistics from a meta-analysis of European-ancestry individuals (N = 1,025,550) which utilized genomic structural equation modeling (gSEM) [2] to identify a latent factor common to four substance use disorders. Polygenic risk scores (PRS) were generated for the target sample using PLINK 2.0, and chromosome-specific scores were aggregated to produce whole-genome predictors [1,22].

We followed the pipeline described by Young (2023) [4] for our susceptibility analysis. To estimate the influence of direct and indirect genetic effects within the MCTFR cohort, we inferred a Parental PRS ($\delta_{k}$) variable from observed genetic data utilizing the Nagelkerke $R^{2}$ derived with our PRS pipeline. To isolate the “pure” biological signal, the snipar package was employed to partition the total PRS into direct genetic effects ($\delta_{k}$)—representing sum additive effects of an individual’s allele on those individual and non-transmitted coefficients (α), which account for indirect environmental influences.

Using these partitioned values, we calculated biological purity ($\rho_{k}$) to quantify the proportion of standard genotype–phenotype associations attributable to direct biological mechanisms. It was derived by taking the ratio of the direct genetic effect ($\delta_{k}$) to the total population-level association ($\beta_{k}$):

(1) $$\rho_{k}=\frac{\delta_{k}}{\beta_{k}}$$

This parameter serves as a metric for the “clean” genetic signal, where lower values suggest that familial clustering accounts for a greater share of the observed association in standard GWAS. Additionally, a reliability factor, k, representing the proportion of the total within-family genetic variance captured by our specific PRS, was derived using the equation:

(2) $$k=\frac{R_{\delta }^{2}}{h_{f}^{2}}$$

Here, $R_{\delta }^{2}$ represents the variance explained by the partitioned direct genetic effect, and $h_{f}^{2}$ is the inheritance derived from biometric ACE twin models, of which the values for the present study were taken from prior MCTFR research [2,3,4,5]. For example, if k is a small value, PRS explains very little of the heritability. We used these values to derive a Parent DGE ($r_{\delta }$) value, which represents an estimated true similarity between parent’s DGE when controlling heritability, and is helpful for capturing the missing variance of DGEs from noisy PRS modeling. It is derived by adjusting the observed whole-genome parental correlation ($r_{k}$) defined as the Pearson correlation between maternal and paternal scores by the factor k, the fraction of total heritability explained by the PRS model.

(3) $$r_{k}=corr\left(PRS_{paternal\left(i\right)}^{\delta_{k}}PRS_{maternal\left(i\right)}^{\delta_{k}}\right)$$

(4) $$r_{\delta }=\frac{r_{k}}{k+r_{k}\left(1-k\right)}$$

We then took this estimate of true DGE to derive a measurement for total proportion of variance explainable by DGE in population that has reached steady state under a pattern of assortive mating:

(5) $$h_{eq}^{2}=\frac{h_{f}^{2}}{1-r_{\delta }}$$

This is a theoretical heritability measure that accounts for clustering of similar genes in families over generations, which would increase the total additive genetic variance in the population beyond the standard heritability measure $h_{f}^{2}$. For example, if it is increased, it means that non-random mating has taken place to the occurrence of the phenotype beyond direct genetics.

Finally, we derived a measurement for contribution to phenotypic variance from the correlation between IGE and DGE, a measurement forhow much IGE provided by parents is correlated with the DGE inherited by the proband:

(6) $$v_{\eta :\delta }=2\;h_{eq}^{2}\;\alpha_{\delta }$$

Transcriptome-wide association analysis was performed via the FUSION framework [23]. By integrating GWAS results with brain-specific eQTL weights from GTEx v8 [24], we estimated the correlation between genetically predicted gene expression and addiction liability. This approach has been validated for its ability to prioritize high-confidence risk genes in neurological and behavioral traits [10,11]. Final gene-level statistics were subjected to Bonferroni correction to maintain stringent control over the type I error rate across all tissue–trait combinations [25,26].

Precomputed expression weights were obtained from GTEx version 8, restricted to brain tissues relevant to reward processing and executive function. The following tissues were included: Brain cortex, Frontal cortex (Brodmann area 9), Caudate (basal ganglia), Putamen (basal ganglia), Substantia nigra, and Cerebellum. TWAS association tests were conducted separately for each phenotype and chromosome using FUSION association. For each gene with available expression weights in each tissue, FUSION computes a test statistic reflecting the association between predicted gene expression and the phenotype of interest. Analyses were performed genome-wide across all autosomes (chromosomes 1–22). Results were generated per chromosome and subsequently merged to produce a genome-wide TWAS result file for each phenotype–tissue pair.

TWAS results were filtered to retain genes with valid association statistics. Genes with missing or non-numeric TWAS *p*-values were excluded. Five continuous addiction-related phenotypes were tested across six GTEx v8 brain tissue panels, yielding 30 phenotype–tissue combinations. The number of testable transcripts varied by tissue according to the availability of heritable expression models in the GTEx v8/FUSION reference panels. Approximately 5000–6000 transcripts were tested in cortex and frontal cortex, 4500–5500 in caudate and putamen, 7500–8500 in cerebellum, and 2500–3500 in substantia nigra. Multiple-testing correction was performed using a tiered Bonferroni strategy. For each phenotype–tissue pair, a phenotype–tissue-specific Bonferroni threshold was calculated as:

$$P_{threshold,\;phenotype-tissue}=\frac{0.05\;}{N_{genes\;tested\;in\;that\;phenotype-tissue\;analysis}}$$

where N_genes tested in that phenotype–tissue analysis_ represents the number of genes with valid FUSION association statistics for that tissue model. Because approximately 150,000 gene–tissue–phenotype tests were evaluated across the full TWAS experiment, we also applied a conservative experiment-wide Bonferroni threshold:

$$P_{threshold,\;study-wide}=\frac{0.05\;}{N_{total\;valid\;gene-tissue\;phenotype\;tests}}$$

Associations were annotated according to whether they survived phenotype–tissue-specific correction, experiment-wide correction, or both.

Significant TWAS results were aggregated across all phenotypes and tissues. For each gene, associations were summarized across phenotypes and brain regions. To reduce redundancy, for each gene–phenotype pair, the association with the strongest statistical signal (lowest *p*-value) was retained. This allowed identification of genes showing: cross-phenotype associations, tissue-specific effects, convergence across multiple brain regions gene identifiers provided by FUSION (Ensembl gene IDs) were mapped to gene symbols using the Ensembl REST API. When mapping was unavailable, original identifiers were retained. Significant gene–phenotype associations were visualized using a bubble plot. In this representation: The *x*-axis denotes phenotype, the *y*-axis denotes gene symbol, bubble size is proportional to −log10(TWAS *p*-value), bubble color represents brain tissue.

*p*-values were additionally annotated directly on the plot to facilitate interpretation of effect magnitude. This visualization enabled identification of genes showing cross-phenotype significance and tissue-specific enrichment patterns. All TWAS analyses were conducted using the FUSION software (Github commit 9346c12) package. Data processing and visualization were performed using Python (3.9) (including pandas, numpy, and matplotlib) and R studio (2024.12.1+563) where appropriate. Analyses were run on a macOS environment using command-line scripting.

Computational tasks were executed on an Ubuntu desktop environment using a combination of custom R-based scripts, GEMMA toolset for LMM associations, PLINK v1.9 for LD clumping (r^2^ ≥ 0.1, ±250 kb window) to identify independent lead variants, SNipar for intergenerational risk partitioning, and the FUSION framework for transcriptomic integration. Additional data processing and visualizations were conducted using Python (pandas, numpy, and matplotlib).

During manuscript preparation, Gemini 3.1 Pro (Google) and ChatGPT 5.5 Thinking (OpenAI) were used as assistive tools for drafting technical descriptions, improving code readability, troubleshooting R studio (2024.12.1+563)/Bash (5.0.17)/Python (3.9) scripts, and generating preliminary narrative summaries of statistical outputs. These tools were not used to generate raw data, modify primary genotype or phenotype files, perform autonomous statistical analyses, or make final scientific conclusions.

**Figure 1:**
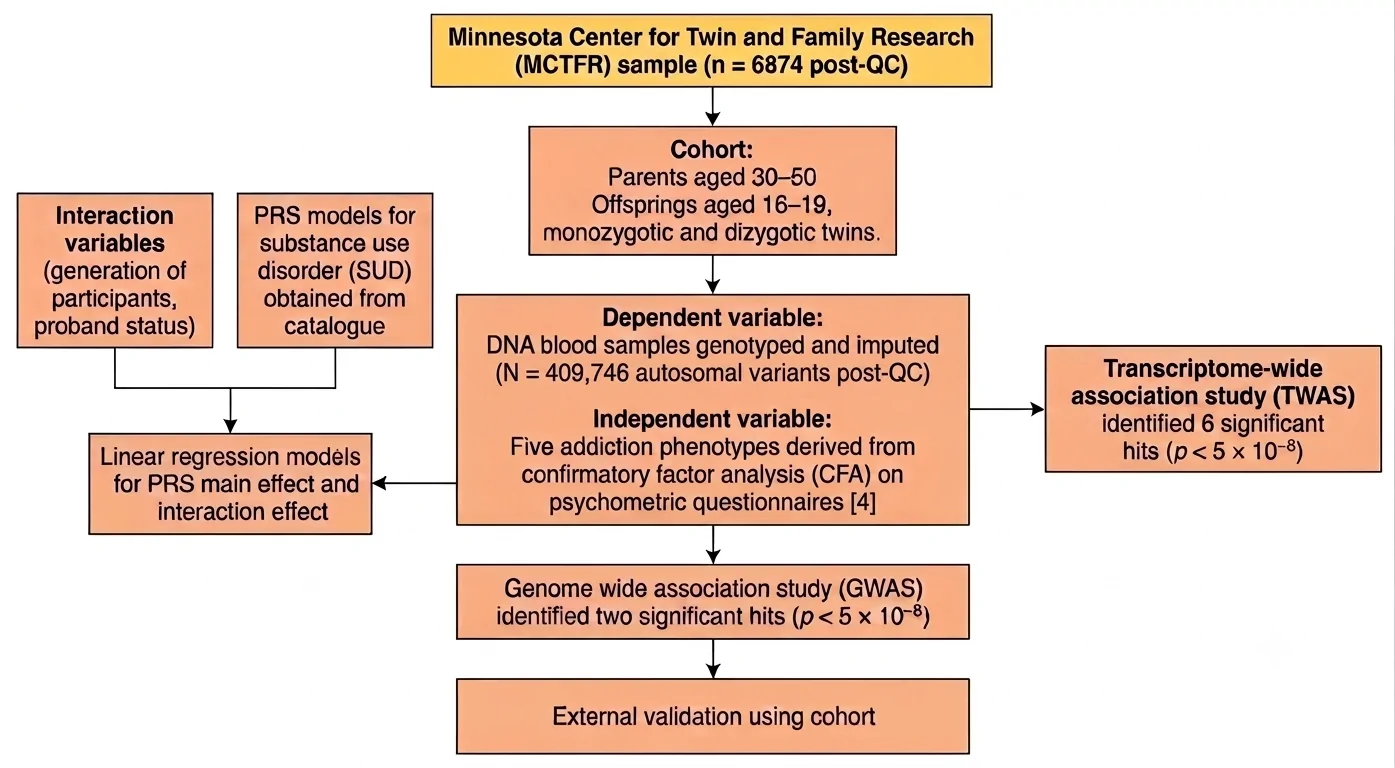
Workflow of the present study.

To reduce the risk of AI-introduced errors, all AI-assisted code was manually reviewed by the authors before execution. Resulting outputs were checked against expected file structures, software logs, summary statistics, and downstream tables. Biological interpretations generated with AI assistance were treated as preliminary hypotheses and revised against the primary literature. The final manuscript text, tables, figures, statistical outputs, and interpretation were reviewed and approved by the authors. See Figure 1. for the schematic of the present study.

## 3. Results

**Figure 2:**
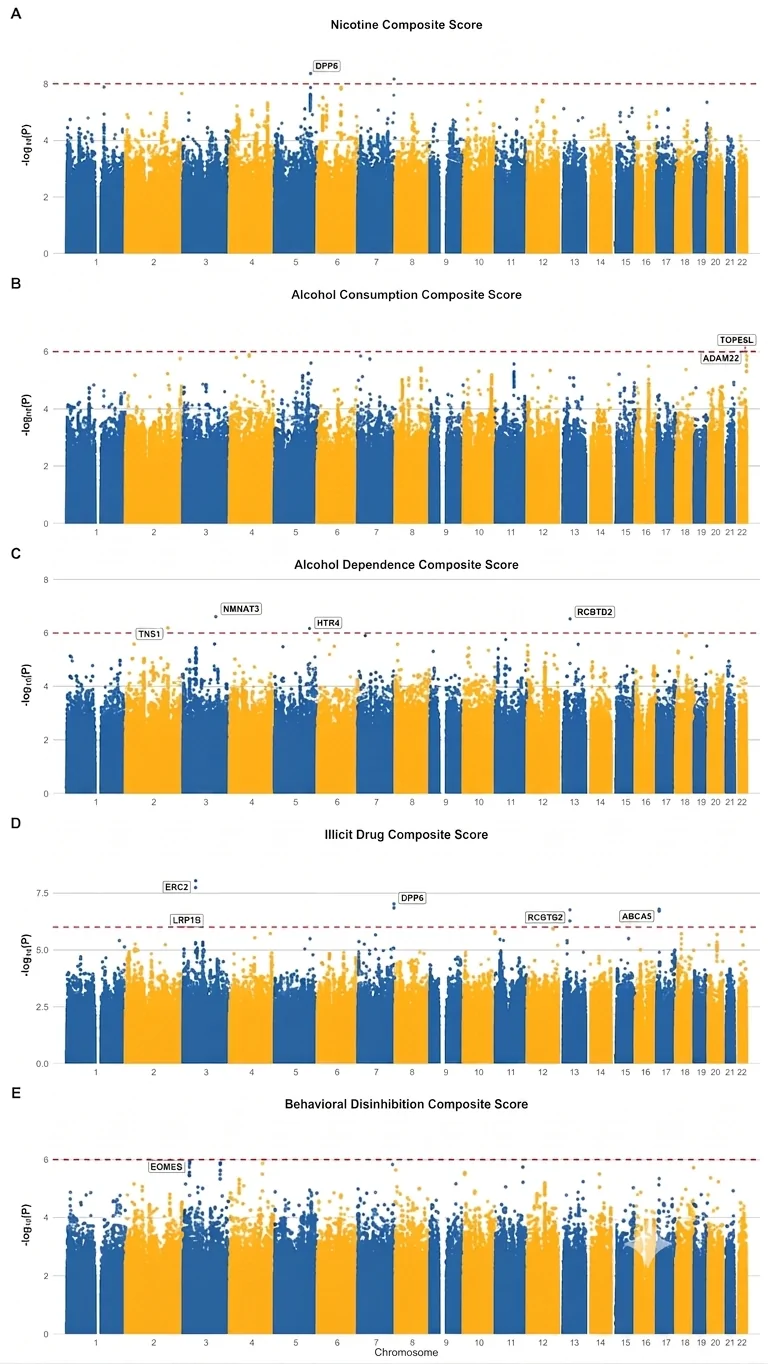
Manhattanplotsof GWAS of SNPs’ main effect on all five phenotypes (composite scores) of addiction: (**A**) nicotine; (**B**) alcohol consumption; (**C**) alcohol dependence; (**D**) illicit drug; (**E**) behavioral disinhibition. The red dashed line represents the genome-wide suggestive significance threshold (*p* = 5 × 10^−6^).

Across all tested traits, 82 independent lead variants reached suggestive significance (*p* < 5 × 10^−6^) while two met the GWAS-standard significance level, (*p* < 5 × 10^−8^). For Manhattan plot visual representation of this finding, see Figure 2. For the top 15 by significance variants (Table 1). Most of these variants reside in non-coding regions, predominantly acting as modifiers via intron or intergenic variation. Of the ones that met suggestive levels, several of interest are rs1368737, an intron variant mapped to the cell adhesion molecule 2 gene CADM2 previously implicated in body mass index, SPC25 gene rs540524 with a minor allele frequency of 0.3896, which prior studies have linked to fasting glucose and glycated hemoglobin levels. Functional annotation via VEP revealed that the majority of these lead variants were non-coding, with 43% located in intergenic regions and 37% within intronic regions (e.g., CLSTN2, NBPF21P). Notable concentrations of risk were observed in the ERC2/EOMES neighborhood on chromosome 3 and the DPP6 locus on chromosome 7, which demonstrated pleiotropic associations across multiple drug and behavioral domains.

The strength and number of associations varied by phenotype: nicotine use showed a limited number of significant loci, with a prominent signal on chromosome 7 (Figure 2A). Alcohol consumption demonstrated comparatively weaker associations, with few loci exceeding the significance threshold (Figure 2B). Alcohol dependence also showed several significant loci, notably on chromosomes 2, 3, 5, and 13 (Figure 2C). Illicit drug use demonstrated the most robust signal, with multiple loci exceeding genome-wide significance, particularly on chromosomes 3, 7, 13, and 17 (Figure 2D). Behavioral disinhibition exhibited a more modest signal, with a single locus reaching the significance threshold on chromosome 3 (Figure 2E). Across all phenotypes significant loci were distributed across multiple chromosomes, suggesting a polygenic architecture underlying substance use-related traits. One variant demonstrated suggestive associations across all five domains, defined as variants with *p* < 1 × 10^−5^ in at least two phenotypes, not previously cataloged. (Table 2).

**Figure 3:**
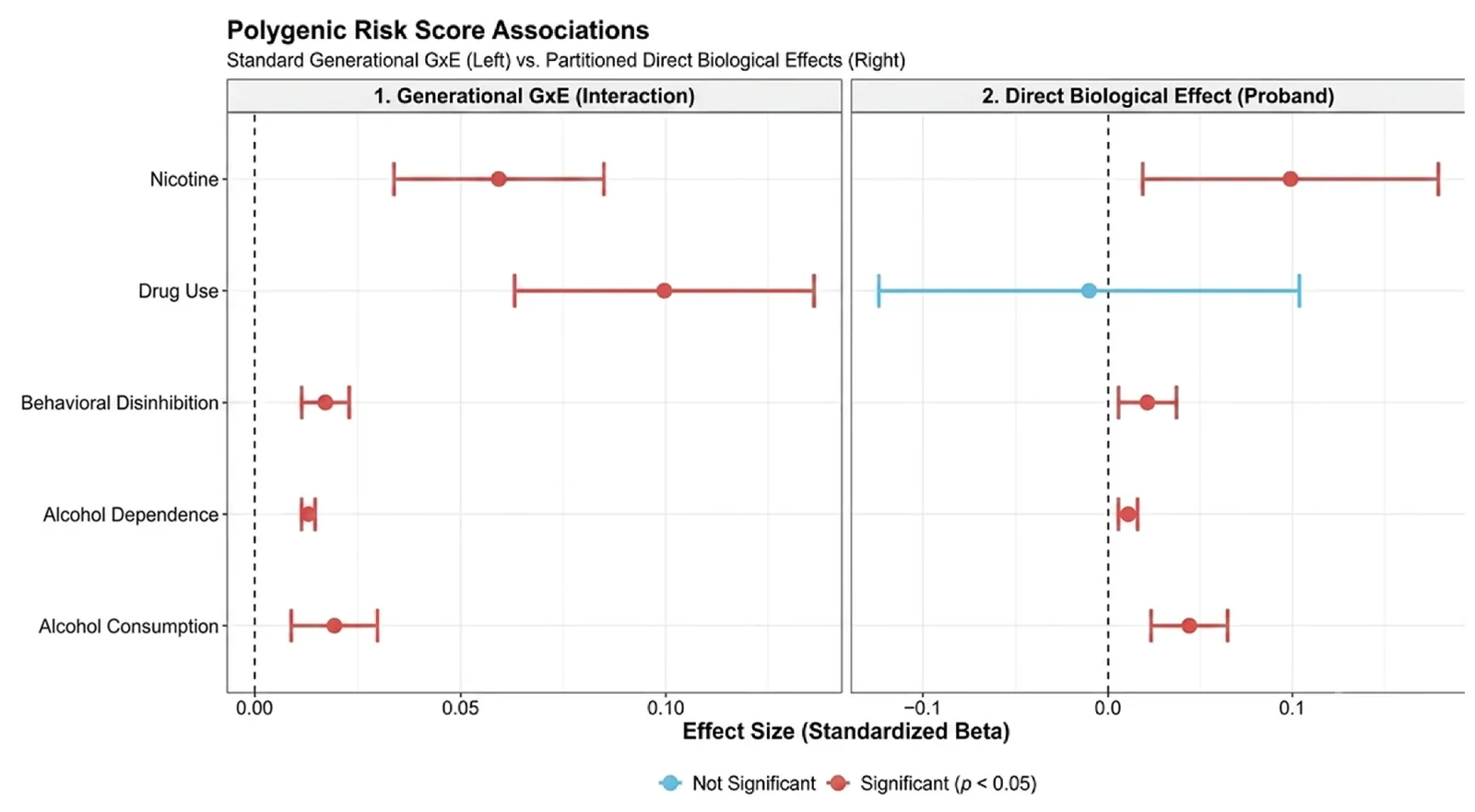
Standardized beta coefficients for the polygenic risk score (PRS) associations across five behavioral phenotypes. In the standard model (**left panel**), we observed a significant Gen × Cohort interaction for all traits, indicating that the penetrance of polygenic liability is significantly greater in the younger generation. Crucially, within-family partitioned models (**right panel**) confirmed that these associations represent direct genetic effects ($\beta_{proband}$), remaining significant while controlling parental PRS.

To calculate an individual’s total genetic liability to a trait, we utilized polygenic risk scores (PRS) after clumping, thus aggregating the small effects of the many variants found. (Figure 3).

Significant direct genetic effects were observed across all five domains, with the strongest biological signal identified for drug use ($\delta_{k}$ = 0.146; Table 3). The calculated biological purity ($\rho_{k}$) is the ratio of direct effect estimated using within-family cohort to population effects effect size estimated using traditional GWAS cohort. It ranged from 59.8% to 68.3%, which means that the remaining 32–40% is attributable to IGE or AM inflation rather than direct biological inheritance.

Our estimated parental genotype correlation (similarity between mother and father’s genetic profile PRS scores) $r_{k}$ was modest (8.4%, Table 3). This value is fundamentally attenuated because the PRS captures only a fraction of the total heritability (Equation (2)). To investigate this attenuation, our estimated Parent DGE ($r_{\delta }$) revealed a much higher correlation, up to 71.9–99.4% similar between the underlying DGEs. Consequently, this high measurement resulted in estimate for equilibrium heritability ($h^{2}eq$) mathematically exceeding the total phenotypic variance ($v_{n:\delta }$). Our baseline heritability ($h^{2}f$) ranged from 0.30 to 0.50, so this would mean that original heritability should be expanded on top of this by over 100% when accounting for assortative mating resulting in a non-randomly selected population. This result was nonsensically high, likely due to our high value. We think this may be due to (1) most likely overestimated heritability in the denominator or (2) underestimated PRS effect size in the numerator (Equations (2), (4) and (5)).

Despite these extreme estimates, the consistent positive directionality of our result still suggests a significant assortative mating effect. The variance contribution from IGE:DGE correlation ($v_{n:\delta }$) is also consistently negative across all domains, ruling out IGEs. In summary, this indicates that the observed GWAS shrinkage can be explained by the statistical artifacts of non-random mating, with no evidence for positive IGE (genetic nurture) influencing these phenotypes.

**Figure 4:**
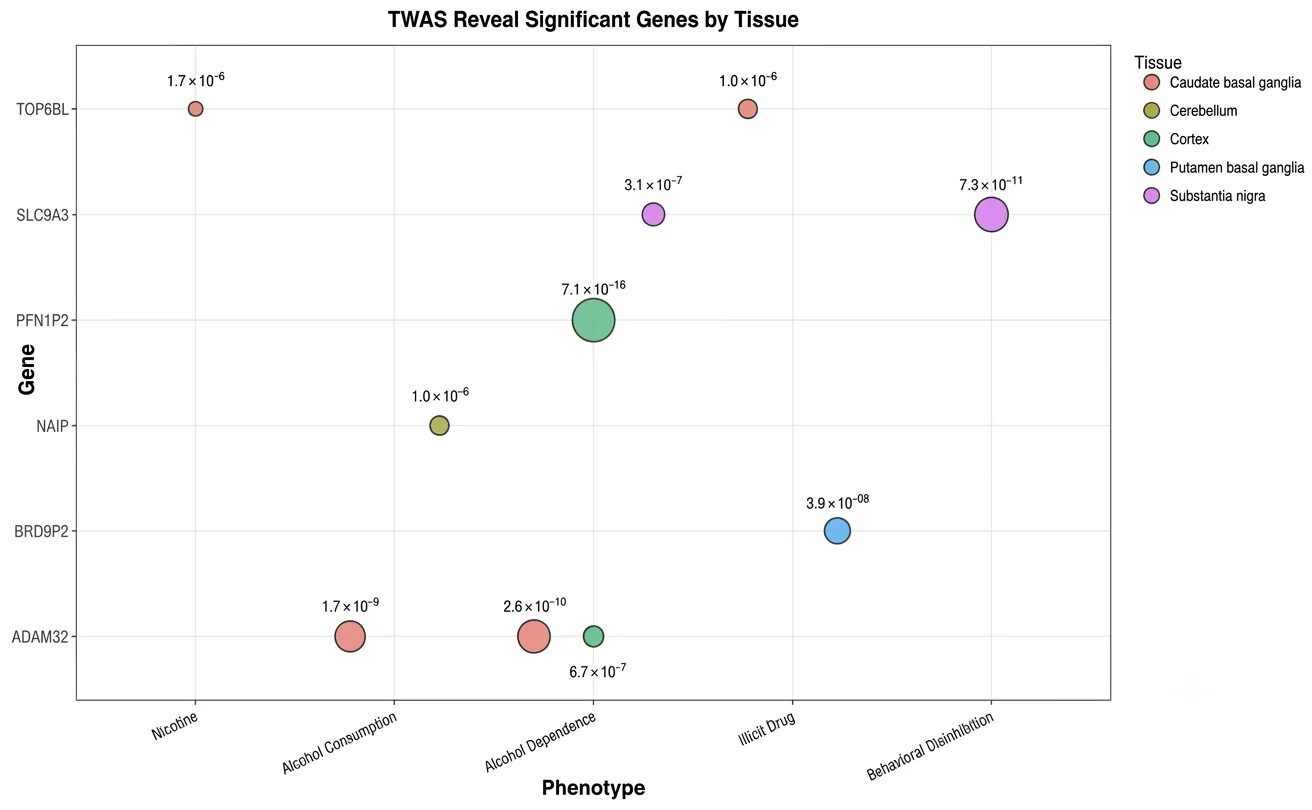
Candidate TWAS associations across selected GTEx v8 brain tissues. Bubble plot showing phenotype–gene associations identified by FUSION TWAS across addiction-related phenotypes and brain tissue panels. The *x*-axis indicates phenotype, the *y*-axis indicates gene symbol, bubble size corresponds to ($-\log_{10}(P)$), and bubble color indicates brain tissue. TWAS associations are shown for candidate genes passing phenotype–tissue-level correction, with experiment-wide Bonferroni status reported in Table 4.

Our TWAS analysis identified several candidate gene–phenotype associations across brain tissues relevant to reward processing, habit formation, and executive function (Figure 4; Table 4). These associations should be interpreted as gene-prioritization signals rather than definitive evidence of biological mechanism. Genetically predicted expression of ADAM32 was associated with alcohol dependence and behavioral disinhibition in the caudate, while TOP6BL was associated with alcohol consumption and illicit drug use in the caudate. SLC9A3 was associated with nicotine use and behavioral disinhibition in the substantia nigra, and PFN1P2 showed the strongest transcriptomic association with behavioral disinhibition in cortical tissue. Additional tissue-specific signals included NAIP in cerebellum for alcohol dependence and BRD9P2 in putamen for illicit drug use.

The known or proposed biological functions of these genes, including roles in synaptic plasticity, ion transport, apoptosis, chromatin regulation, and cytoskeletal dynamics, provide biologically plausible directions for future investigation [27,28,29,30,31,32,33,34]. However, TWAS does not establish that altered expression of these genes causes addiction-related phenotypes, nor does it confirm the precise tissue, cell type, developmental stage, or direction of biological effect. Therefore, these findings are best viewed as hypotheses for downstream validation using colocalization, fine mapping, conditional analyses, cell-type-specific expression data, and experimental functional studies [10,11,25,26].

Candidate TWAS associations across striatal and frontal cortical structures. TWAS associations showed brain-region-specific patterns in tissues relevant to reward processing and executive function. Predicted expression of ADAM32 in the caudate was associated with alcohol dependence and behavioral disinhibition, while TOP6BL in the caudate was associated with alcohol consumption and illicit drug use. In the substantia nigra, SLC9A3 was associated with nicotine use and behavioral disinhibition. The strongest transcriptomic association was observed for PFN1P2 in cortical tissue for behavioral disinhibition. These findings nominate candidate genes and tissues for future functional study but do not establish causal mechanism, tissue-specific biological effect, or shared causal variants with GWAS loci.

Similarly, *TOP6BL* expression in the caudate was associated with both Alcohol Consumption and Illicit Drug use. While *TOP6BL* (Topoisomerase VI Binding Protein Like) is classically understood to mediate DNA double-strand breaks during meiosis, emerging neurobiological evidence demonstrates that rapid, activity-dependent DNA double-strand breaks in neurons are a fundamental mechanism for early-response gene transcription during learning and memory formation. Differential regulation of *TOP6BL* in the striatum may therefore alter experience-dependent transcriptional plasticity following drug exposure.

Ion Homeostasis and Dopaminergic Tone. In the substantia nigra, a primary source of dopaminergic projections, *SLC9A3* expression, was robustly associated with nicotine use and behavioral disinhibition [27]. *SLC9A3* encodes a sodium/hydrogen exchanger crucial for maintaining intracellular pH and ion homeostasis. Because dopaminergic neuron firing and synaptic vesicle loading are highly dependent on precise transmembrane ion gradients, genetically driven variations in *SLC9A3* expression may fundamentally alter baseline dopaminergic tone and sensitivity to psychostimulants like nicotine.

Tissue-Specific and Non-Coding Regulatory Mechanisms. In addition to cross-trait loci, our study identified highly localized, trait-specific signals. *NAIP* (NLR Family Apoptosis Inhibitory Protein) was significantly associated with alcohol dependence specifically in the cerebellum. Given that cerebellar atrophy is a hallmark of chronic alcohol toxicity, variations in the expression of *NAIP* (a potent neuroprotective and anti-apoptotic factor) may modulate cellular vulnerability to alcohol-induced neurodegeneration.

Finally, our TWAS highlighted the regulatory importance of pseudogenes in addiction susceptibility. PFN1P2 (Profilin 1 Pseudogene 2) in the cortex was our most significant overall hit, profoundly linked to behavioral disinhibition. The parent gene, Profilin 1, is a master regulator of actin cytoskeleton dynamics and dendritic spine morphology. Pseudogenes frequently act as epigenetic modulators or competing endogenous RNAs for their parent genes; thus, PFN1P2 may indirectly govern the dendritic spine remodeling in the cortex necessary for top-down executive control. Similarly, the association of BRD9P2 (Bromodomain Containing 9 Pseudogene 2) with illicit drug use in the putamen highlights a potential epigenetic mechanism, as bromodomain proteins are critical “readers” of histone acetylation involved in the long-term transcriptional changes that solidify compulsive drug-seeking behaviors. A summary of the functional gene by brain tissue and phenotype is shown in Table 4.

Across the TWAS analysis, 5 continuous addiction-related phenotypes were tested across 6 GTEx v8 brain tissue panels, yielding 30 phenotype–tissue combinations and approximately 150,000 total gene–tissue–phenotype tests. This resulted in an experiment-wide Bonferroni threshold of (*p* < 3.3 × 10^−7^). Several TWAS associations remained significant after this conservative experiment-wide correction, including PFN1P2 in cortex for behavioral disinhibition (*p* = 7.12 x 10^−16^, Z = +8.07), SLC9A3 in substantia nigra for nicotine use (*p* = 7.26 × 10^−11^, Z = −6.52), and ADAM32 in caudate for behavioral disinhibition (*p* = 2.57 × 10^−10^, Z = +6.32). Other associations are reported as phenotype–tissue-level candidate for TWAS findings and interpreted cautiously.

Rather than providing definitive locus-level convergence between GWAS and TWAS signals, our analyses offer complementary variant-level and transcriptome-level evidence for addiction-related genetic architecture. (Table 5, Appendix A) At the GWAS level, suggestive lead variants were primarily non-coding and mapped to loci including PLXNB2, CLSTN2, RB1, DPP6-region variants, and other intergenic or intronic regions. At the transcriptomic level, TWAS prioritized several brain-region-specific genes, including ADAM32, TOP6BL, SLC9A3, PFN1P2, NAIP, and BRD9P2. These TWAS signals may implicate biologically relevant neural circuits, particularly striatal and frontal cortical regions, but they should not be interpreted as evidence that the same causal variants underlie both GWAS and TWAS associations without formal colocalization, fine mapping, shared credible-set analysis, or LD-based locus overlap.

**Table 5:** Master summary of genomic and transcriptomic analysis for addiction.

Phenotype	Clumped Locus	Lead rsID	Position (BP)	Physical Consequence	GWAS Beta (SE)	GWAS P	Functional Gene	TWAS P	TWAS Z	Circuit/Function
Nicotine	DPP6	rs190049611	7:153,433,844	Intergenic	0.18 (0.03)	9.36 × 10^−8^	SLC9A3	7.26 × 10^−11^	−6.52 (↓)	Sub. Nigra/pH
Alc. Cons.	ADAM32	rs140030949	22:50,725,237	PLXNB2 Downstream	−0.10 (0.03)	8.56 × 10^−7^	TOP6BL	1.72 × 10^−6^	−4.78 (↓)	Caudate/Plasticity
Alc. Dep.	ERC2	rs62267967	3:139,986,727	CLSTN2 Intron	0.26 (0.07)	2.50 × 10^−7^	ADAM32	1.67 × 10^−9^	+6.03 (↑)	Striatal Habit Hub
Alc. Dep.	SLC9A3	rs4530746	5:148,871,502	CSNK1A1 Downstream	0.11 (0.04)	6.91 × 10^−7^	NAIP	1.01 × 10^−6^	+4.89 (↑)	Cerebellum/Prot.
Drug Use	ERC2	rs141943748	3:54,112,290	Intergenic	0.38 (0.07)	8.77 × 10^−9^	BRD9P2	3.85 × 10^−8^	−5.50 (↓)	Putamen/Epigenetics
Disinhibition	ERC2	rs140880829	3:36,662,400	NBPF21P Intron	0.16 (0.05)	1.08 × 10^−6^	PFN1P2	7.12 × 10^−16^	+8.07 (↑)	Frontal Cortex/Actin
Disinhibition	Pleiotropic	—	—	—	—	—	ADAM32	2.57 × 10^−10^	+6.32 (↑)	Executive Control
Disinhibition	Pleiotropic	486599:20:00	3:29,195,780	Intergenic	−0.05 (0.02)	9.98 × 10^−7^	SLC9A3	3.10 × 10^−7^	−5.12 (↓)	Sub. Nigra/Ions

Clumped Locus: the genomic region identified by the leading SNP. Physical Consequence: the functional classification of the variant based on its genomic location. GWAS Beta (SE): the effect size and standard error (SE), GWAS P: the statistical significance of the SNP association derived from the linear mixed model, unctional Gene: specific gene prioritized by the TWAS framework, TWAS P: the statistical significance of the association between the phenotype and the genetically regulated gene expression, TWAS Z: the strength and direction of the transcriptomic association, (↑) is higher predicted expression with increased addiction risk, (↓) indicates that lower predicted expression is associated with increased addiction risk, Circuit Function is the anatomic brain region where the gene is significantly expressed and functionally relevant.

## 4. Discussion

### 4.1. Summary of Findings

In our study, we performed genome-wide and transcriptome-wide association analyses across multiple substance use-related phenotypes, uncovering a complex polygenic architecture that spans several domains. By leveraging a modern family-based GWAS toolset, we identified a slightly higher number of significant SNPs than the original 2014 study by McGue et al., though none of these variants passed external validation, a result we attribute to a loss of statistical power following data transformation. We successfully localized an enrichment of TWAS signals within the striatal and frontal brain regions, providing further insight into the biological underpinnings of these traits. Finally, through mathematical modeling of PRS scoring, we demonstrated that large assortative mating effects are at play, whereas we found no evidence of genetic nurture effects.

These findings provide empirical support for transdiagnostic models of addiction, demonstrating a substantial genetic overlap across distinct substance use domains driven by a shared polygenic architecture. This underlying vulnerability maps directly onto the Externalizing Spectrum of the HiTOP framework and aligns with the RDoC Cognitive Systems domain (Inhibitory Control subconstruct).

### 4.2. Novel and Candidate Genes

We identified suggestive signals corresponding to distinct substance use behaviors, including variants mapped to SPC25, CADM2, and RB1 for drug use, CSNK1A1 and PRDM11 for alcohol dependence, PLXNB2 for alcohol consumption, PYHIN1 for nicotine use, and CNTNAP2 for generalized behavioral disinhibition. Our significant signals did not correlate to any previously defined traits or genes.

While some identified genes have prior associations with broader complex traits, such as the established link between CADM2 and body mass index, others represent potentially novel candidates for specific substance use biology. For example, variants mapped to SPC25 for drug use and PRDM11 for alcohol dependence have prior associations with systemic metabolic and endocrine markers, including fasting glucose, glycated hemoglobin, and thyroid stimulating hormone levels. Furthermore, we identified strong neurodevelopmental candidates such as CNTNAP2 associated specifically with behavioral disinhibition. Because these variants predominantly act as non-coding modifiers, these findings provide a biological basis for future studies investigating their regulatory roles in neural circuitry.

### 4.3. Biological Interpretation

Our findings are consistent with a hypothesis in which addiction-related phenotypes involve genetically regulated expression differences in brain regions related to reward learning, habit formation, and executive control. Candidate TWAS signals in striatal tissues, including ADAM32, TOP6BL, and BRD9P2, may reflect biological pathways relevant to synaptic plasticity, activity-dependent transcription, and chromatin regulation, while the cortical PFN1P2 signal may implicate pathways related to cytoskeletal regulation and neuronal structure [29,32,34]. However, these interpretations remain hypothesis-generating. TWAS does not demonstrate that these genes directly cause addiction-related phenotypes or that they mechanistically regulate the implicated neural circuits.

The presence of TWAS associations in the caudate, putamen, and substantia nigra is consistent with prior evidence implicating striatal and dopaminergic circuitry in reward learning, reinforcement, and motivational salience [27,28]. Associations observed in frontal cortical regions are also compatible with the role of executive function and inhibitory control in behavioral disinhibition. The presence of cross-phenotype candidate genes, including ADAM32 and SLC9A3, supports the possibility of shared transcriptomic liability across substance-use traits, but these findings require formal colocalization, fine mapping, and functional validation before causal or tissue-specific mechanisms can be inferred [10,11,25,26,35].

To clarify the functional mechanism underlying our top transcriptomic signal, the prominent association between the pseudogene *PFN1P2* and behavioral disinhibition likely operates via non-coding regulatory pathways rather than canonical translation. *PFN1P2* (Profilin 1 Pseudogene 2) is a transcribed pseudogene that exhibits functional characteristics highly analogous to long non-coding RNAs (lncRNAs). Rather than translating into a functional protein, transcribed pseudogenes frequently act as competing endogenous RNAs (ceRNAs), or “microRNA sponges.” In this regulatory role, *PFN1P2* transcripts competitively bind specific microRNAs, thereby preventing those microRNAs from degrading the mRNA of its parental gene, *PFN1*, or other critical synaptic transcripts within the frontal cortex. Disruption of this non-coding regulatory network provides a plausible molecular mechanism for altered cortical excitability and the subsequent impairment of top-down cognitive control observed in behavioral disinhibition.

### 4.4. Genetic Susceptibility

Standard GWAS pipelines typically generate population effects that conflate direct biological inheritance with the confounding influences of indirect genetic effects (IGEs) and assortative mating (AM). By failing to partition these components, traditional models risk overestimating the functional impact of individual alleles and mischaracterizing a trait’s underlying genetic architecture as purely biological rather than a mixture of nature and nurture. We addressed this by applying the SNipar framework to five substance use domains, identifying significant direct genetic effects (DGEs) across all phenotypes.

Our analysis revealed that biological purity ($\rho_{k}$) ranged from 59.8% to 68.3% (Table 3), indicating that a substantial additive effect remains after controlling familial inflation. However, our estimation procedure produced extreme results for several domains, including equilibrium heritability ($h_{eq}^{2}$) estimates significantly above 1—most notably reaching 83.3 for alcohol dependence. The equilibrium heritability estimate is so high because our estimate of the correlation between parents’ DGE components ($r_{\delta }$) is extreme, reaching up to 0.994 (Table 3). We think that this derives from the fact that while the correlation between parents’ observed PRS ($r_{k}$) was modest at 8.4% (Table 3), the PRS explains only a fraction of the heritability assuming a randomly mating population. As hypothesized in the paper from Miller 2023, who also encountered this problem in their simulation case, these implausible results likely stem from the overestimation of heritability by traditional twin studies [4]. Despite these points of model saturation, the directionality of our results remains clear. The calculated contribution to phenotypic variance from the DGE-IGE correlation ($v_{\eta :\delta }$) was consistently negative across all domains (e.g., −26.5 for alcohol dependence; Table 3).

This finding indicates no evidence for positive IGEs on substance use, suggesting that the observed GWAS shrinkage is explained by the statistical artifacts of assortative mating rather than environmental nurture.

### 4.5. Limitations

Our discovery sample size of 6874 individuals (pruned from 7188 after quality checks) is relatively small for modern genomic discovery. This limited statistical power, combined with the reassessment of a previously analyzed cohort [15], likely constrained our ability to detect variants of small effect and explain the absence of many significant signals. Relying exclusively on European ancestry populations for both our discovery sample and linkage disequilibrium reference panels limits the generalizability of our findings to other ethnic groups.

The continuous composite phenotypes of the MCTFR cohort were constructed using a hierarchical factor analytic approach that integrates the frequency, quantity, and formal clinical symptoms of substance dependence into a single continuous liability score [14,33]. Crucially, this framework does not reflect sporadic use. It embeds severe clinical variation by incorporating diagnostic symptoms of DSM-III-R and DSM-IV Nicotine, Alcohol, and Illicit Drug Dependence. While these data were collected under legacy diagnostic manuals, this dimensional structure mirrors the paradigm shift of the DSM-5 toward continuous severity scales. It also serves as an empirical representation of the HiTOP Externalizing Spectrum and aligns with the RDoC Cognitive Systems domain (Inhibitory Control).

However, replicating such transdiagnostic phenotypes presents a challenge for external validation, as few twin cohorts share this exact dimensional architecture. This issue of phenotypic mismatch is illustrated by our lookup within the NIDA cohort. Although both cohorts share roots in legacy DSM-III R, DSM-4 diagnostic criteria for phenotype, none of our 82 independent suggestive variants survive the strict Bonferroni threshold. Only a single variant (1:223688138) achieved nominal replication (Supplementary Table S1). As the validation was performed using a non-directional quasi-likelihood score test (MFQLS), we could not evaluate directional concordance between the two cohorts. We acknowledge that dichotomizing such continuous data to match traditional diagnostic criteria introduces a catastrophic loss of statistical power, though this constraint was necessary due to the scarcity of independent cohorts sharing a dimensional design [36,37,38,39]. This study highlights the pressing need to shift the field toward harmonized dimensional phenotyping, which will be critical for future biobanks to adequately power association studies.

Finally, TWAS identifies statistical correlations but does not establish strict causality, as observed signals may arise from linkage disequilibrium rather than direct functional gene effects [26]. Because we did not perform formal colocalization or fine mapping analyses, we cannot definitively conclude that the genomic and expression signals share the exact same causal variant. Furthermore, relying on bulk tissue expression weights from GTEx version 8 [24] may fail to capture cell-type-specific patterns, developmental stage effects, or context-dependent gene regulation unique to acute substance exposure.

Although TWAS can help prioritize genes whose genetically regulated expression is associated with addiction-related phenotypes, these associations do not establish that the TWAS gene and the nearest GWAS locus share the same causal variant. We did not perform formal colocalization, fine mapping, or credible-set overlap analyses; therefore, we avoid interpreting the TWAS results as definitive GWAS–TWAS convergence. Future work should integrate colocalization methods, tissue-specific eQTL fine mapping, and LD-based locus matching to determine whether the observed GWAS and TWAS signals arise from shared causal architecture. Additionally, approaches such as weighted gene co-expression network analysis, cell-type-specific eQTL integration, and expression-coordination testing across striatal and cortical tissues could help determine whether ADAM32 belongs to broader transcriptomic modules associated with addiction-related phenotypes.

## 5. Conclusions

This study supports a model in which addiction vulnerability reflects shared, transdimensional genetic liability with candidate transcriptomic signals in striatal and frontal cortical brain regions. By estimating direct genetic effects [8], this study provides evidence for a measurable inherited component of addiction-related phenotypes, while TWAS nominates candidate genes in striatal and cortical tissues for future functional investigation, such as ADAM32 and TOP6BL which could govern the “bottom-up” drive for habit formation [29]. Furthermore, the cortical PFN1P2 association may reflect a transcriptomic signal relevant to executive control and inhibitory regulation [34]. Crucially, the clustering of these traits within families is shown to be a biological phenomenon magnified by assortative mating [4]. Ultimately, the work argues that continuous, dimensional phenotyping is more accurate than traditional categorical diagnoses, which suffer from a phenotypic sensitivity gap that obscures the polygenic signals underlying addictive behavior.

## Figures and Tables

**Table 1:** Post-clumped GWAS significant SNPs.

Trait	Lead Variant	Position (Chr:BP)	Gene	Consequence	Beta (SE)	*p*-Value (LRT)	Prior Associations	PMID
Drug Use	rs141943748	3:54112290	NA	intergenic_variant	0.38(0.07)	8.77 × 10^−9^	N/A	N/A
Drug Use	rs190049611	7:153433844	NA	intergenic_variant	0.18(0.03)	9.36 × 10^−8^	N/A	N/A
Drug Use	rs141553718	17:10702703	RP11-963H4.5	downstream_gene_variant	0.31(0.06)	1.63 × 10^−7^	N/A	N/A
Drug Use	rs56065157	13:48927587	RB1	intron_variant	0.35(0.07)	1.69 × 10^−7^	N/A	N/A
Drug Use	rs145852361	17:11650097	DNAH9	intron_variant	−0.24(0.05)	1.86 × 10^−7^	N/A	N/A
Alcohol Dependence	rs62267967	3:139986727	CLSTN2	intron_variant	0.26(0.07)	2.50 × 10^−7^	N/A	N/A
Alcohol Dependence	rs143161275	2:180554440	ZNF385B	intron_variant	0.179(0.05)	6.79 × 10^−7^	N/A	N/A
Alcohol Dependence	rs4530746	5:148871502	CSNK1A1	downstream_gene_variant	0.11(0.04)	6.91 × 10^−7^	N/A	N/A
Alcohol Consumption	rs140030949	22:50725237	PLXNB2	downstream_gene_variant	−0.1(0.03)	8.56 × 10^−7^	N/A	N/A
Drug Use	rs540524	2:169756930	SPC25	intron_variant	−0.08(0.02)	9.30 × 10^−7^	Glycated hemoglobin levels; fasting glucose; random glucose levels; fasting blood glucose	34059833; 37679419; 35902682; 33842983
Behavioral Disinhibition	3:29195780	3:29195780	NA	intergenic_variant	−0.05(0.02)	9.98 × 10^−7^	N/A	N/A
Behavioral Disinhibition	rs140880829	3:36662400	NBPF21P	intron_variant,non_coding_transcript_variant	0.16(0.05)	1.08 × 10^−6^	N/A	N/A
Drug Use	12:110132906	12:110132906	NA	intergenic_variant	0.29(0.06)	1.21 × 10^−6^	N/A	N/A
Alcohol Dependence	rs75057314	18:42870971	SLC14A2	intron_variant	0.20(0.05)	1.25 × 10^−6^	N/A	N/A
	rs141120554	7:31883612	PDE1C	downstream_gene_variant	0.022(0.08)	1.26 × 10^−6^	N/A	N/A

Post-clumping GWAS significant SNPs. Top 15 by *p*-value independent lead loci associated with substance use phenotypes. No variants passed external validation with confirmatory cohort after Bonferroni correction.

**Table 2:** Pleiotropic SNPs.

RSID	Chr:Position	Phenotypes	Minimum *p*-Value
rs856084	1: 158915141	3, 5	1.7 × 10^−6^
rs56065157	13:48927587	3, 4	1.69 × 10^−7^
rs145852361	17:11650097	4, 5	1.86 × 10^−7^
rs75057314	18:42870971	2, 3	1.25 × 10^−6^
rs142946758	2:36653189	3, 5	2.65 × 10^−6^
rs190049611	7:153433844	1, 4	9.36 × 10^−8^
rs144111968	7:153471363	1, 4	1.42 × 10^−7^

Pleiotropic variant defined as univariate SNPs with *p* < 1 × 10^−5^ in at least two phenotypes.

**Table 3:** Polygenic risk score (PRS) associations.

Phenotype	NTC ($\alpha_{PGI}$)	DGE ($\delta_{k}$)	Purity ($\rho_{k}$)	Parent PRS ($r_{k}$)	Parent DGE ($r_{\delta }$)	Equilibrium ($h_{eq}^{2}$)	IGE:DGE ($v_{n:\delta }$)
Nicotine	0.059 ***	0.099 *	60.7%	8.4%	0.85 (0.06)	2.98 (1.25)	−0.31 (0.47)
Alcohol Consumption	0.019 ***	0.044 ***	68.1%	8.4%	0.97 (0.03)	7.9 (7.47)	−3.76 (3.99)
Alcohol Dependence	0.013 ***	0.021 *	59.8%	8.4%	0.99 (0.02)	83.3 (369)	−26.5 (121.19)
Drug Use	0.063 ***	0.147 ***	68.3%	8.4%	0.72 (0.09)	1.25 (0.44)	−0.16 (0.023)
Behavioral Disinhibition	0.020 ***	0.045 ***	67.5%	8.4%	0.98 (0.02)	17.9 (25.23)	−8.52 (13.12)

The direct effect ($\delta_{k})$ quantifies the causal biological influence of an individual’s inherited alleles (DGE). Indirect/NTC ($\alpha_{PGI})$ represents the average non-transmitted coefficient (NTC). Biological purity ($\rho_{k})$ is the ratio of the PRS model that is attributable to DGE. Parent PRS ($r_{k}$) denotes the raw correlation between the parent’s measured PRS. Parent DGE $r_{\delta }$ characterizes the inferred correlation between parents’ underlying direct genetic components. *p* value significance denoted by ***: *p* < 0.001 *: *p* < 0.05. In parenthesis is standard error.

**Table 4:** Candidate TWAS associations by brain tissue and phenotype (Bonferroni corrected).

Phenotype	Brain Tissue	Gene Symbol	Ensembl ID	Z-Score	*p*-Value
Nicotine	Substantia nigra	SLC9A3	ENSG00000066230	−6.52	7.26 × 10^−11^
Alcohol Consumption	Caudate basal ganglia	TOP6BL	ENSG00000173715	−4.78	1.72 × 10^−6^
Alcohol Dependence	Caudate basal ganglia	ADAM32	ENSG00000197140	6.03	1.67 × 10^−9^
Alcohol Dependence	Cerebellum	NAIP	ENSG00000249437	4.89	1.01 × 10^−6^
Illicit Drug	Putamen basal ganglia	BRD9P2	ENSG00000249908	−5.5	3.85 × 10^−8^
Illicit Drug	Caudate basal ganglia	TOP6BL	ENSG00000173715	−4.89	9.98 × 10^−7^
Behavioral Disinhibition	Cortex	PFN1P2	ENSG00000270392	8.07	7.12 × 10^−16^
Behavioral Disinhibition	Caudate basal ganglia	ADAM32	ENSG00000197140	6.32	2.57 × 10^−10^
Behavioral Disinhibition	Substantia nigra	SLC9A3	ENSG00000066230	−5.12	3.10 × 10^−7^
Behavioral Disinhibition	Cortex	ADAM32	ENSG00000197140	4.97	6.69 × 10^−7^

## Data Availability

The R, Bash, and Python scripts used for genotype quality control, GWAS execution, LD clumping, PRS construction, TWAS analysis, post-processing, and figure generation are publicly available at: https://github.com/jared8844/code-for-addiction-paper/tree/main (accessed on 14 July 2026). The repository includes the analysis workflow, relevant command-line calls, software versions, package dependencies, and available computational logs. Final tables were regenerated from the final analysis of outputs and manually checked for consistency with the manuscript.

## Supplementary Materials

The following supporting information can be downloaded at: https://www.mdpi.com/article/10.3390/biomedicines14081677/s1, Table S1: GWAS Association Results Cross-Trait Master Sheet.

## Appendix A

**Table A1:** Biological function of significant gene hits.

Gene	Biological Function and Reference
*SLC9A3*	Regulates intracellular pH and dopamine tone [27]
*DPP6*	Modulates dendritic excitability via K+ channels [28]
*TOP6BL*	Mediates activity-dependent DNA breaks [29]
*ADAM32*	Axonal guidance and synaptic plasticity
*NAIP*	Inhibits apoptosis and alcohol-induced toxicity [30]
*ERC2*	Organizes presynaptic active zone signaling [31]
*BRD9P2*	Epigenetic reader of lysine acetylation [32]
*ABCA8*	Regulates CNS lipid and cholesterol transport [33]
*PFN1P2*	Regulates actin cytoskeleton and spine density [34]

Gene name and associated function per literature.
